# Estimating Stair Running Performance Using Inertial Sensors

**DOI:** 10.3390/s17112647

**Published:** 2017-11-17

**Authors:** Lauro V. Ojeda, Antonia M. Zaferiou, Stephen M. Cain, Rachel V. Vitali, Steven P. Davidson, Leia A. Stirling, Noel C. Perkins

**Affiliations:** 1 Department of Mechanical Engineering, University of Michigan, Ann Arbor, MI 48109, USA; smcain@umich.edu (S.M.C.); vitalir@umich.edu (R.V.V.); stevepd@umich.edu (S.P.D.); ncp@umich.edu (N.C.P.); 2 Department of Orthopedic Surgery, Rush University Medical Center, Chicago, IL 60612, USA; antonia_zaferiou@rush.edu; 3 Department of Aeronautics and Astronautics, Massachusetts Institute of Technology, Boston, MA 02139, USA; leia@mit.edu

**Keywords:** wearable sensors, inertial measurement units, motion tracking, human performance, stair running

## Abstract

Stair running, both ascending and descending, is a challenging aerobic exercise that many athletes, recreational runners, and soldiers perform during training. Studying biomechanics of stair running over multiple steps has been limited by the practical challenges presented while using optical-based motion tracking systems. We propose using foot-mounted inertial measurement units (IMUs) as a solution as they enable unrestricted motion capture in any environment and without need for external references. In particular, this paper presents methods for estimating foot velocity and trajectory during stair running using foot-mounted IMUs. Computational methods leverage the stationary periods occurring during the stance phase and known stair geometry to estimate foot orientation and trajectory, ultimately used to calculate stride metrics. These calculations, applied to human participant stair running data, reveal performance trends through timing, trajectory, energy, and force stride metrics. We present the results of our analysis of experimental data collected on eleven subjects. Overall, we determine that for either ascending or descending, the stance time is the strongest predictor of speed as shown by its high correlation with stride time.

## 1. Introduction

We present a method for using inertial measurement units (IMUs) to measure the kinematics and performance of stair running. Running on stairs is a mechanically challenging task. Stair ascent (both walking and running) challenges the body to achieve center of mass translation forward and upward against gravity (repeatedly generating upward ground reaction forces larger than the downward bodyweight force). Therefore, studying stair ascent can provide insights into an individual's aerobic conditioning [1], athletic strength and lower extremity power [2], and performance [3]. Stair descent, in contrast, challenges the body to achieve the desired forward and downward trajectory while controlling and leveraging the assistance of gravity. Therefore, stair descent performance is often studied in clinical populations to assess the level of lower extremity joint stability and control [4]. Furthermore, each footfall needs to land on the relatively small surface of each step, therefore, successful performance of both stair ascent and decent require body coordination across multiple body segments in order to avoid trips or falls. Overground running has been studied extensively from different points of view [5,6]; a detailed review of early research being provided by Novacheck [7]. On the other hand, in-depth biomechanical analysis of stair running has been limited by inadequate biomechanical tracking tools. Optical-based motion capture systems and instrumented walkways, which are commonly used for studying gait, are limited by practical challenges in order to appropriately position cameras for the desired motion capture volume. Consequently, past studies of stair climbing focus on the functional walking pace [8,9,10] and have estimated overall energy expenditure [11], basic timing measures [12], and joint angles [6].

In contrast, we propose using foot-mounted IMUs as a motion capture instrument. Body-worn IMUs enable human motion analysis in outdoor and other contextually-relevant settings (e.g., training facilities, game settings, obstacle courses) and have been used in a wide array of biomechanics applications; see, for example, [13,14,15,16,17,18,19,20]. Our approach uses foot-mounted IMUs to measure the foot kinematic variables (acceleration and angular velocity) during stair running. Doing so enables one to track a large number of steps, to understand transient and steady state running on stairs, and to also deduce performance measures.

IMUs are portable, unobtrusive, and unconstrained (e.g., they do not need external references) motion tracking devices. However, IMU data (and quantities computed therefrom) are affected by several sources of error (e.g., bias instability, scale factor errors, acceleration, and temperature sensitivity) that must be accounted for during motion tracking applications [21]. In this paper, we present specialized algorithms that address these sources of error to estimate the foot trajectory and velocity during stair running. In particular we extend the Zero velocity UPdaTe (ZUPT) algorithm [22], which has been validated to provide accurate foot motions [14,23], by adding additional drift corrections specific to the constraints of stair running (known riser dimensions). We further employ those estimates to deduce metrics for evaluating stair running performance and explore the metrics utilizing experimental data collected on 11 subjects. We hypothesized that the metrics that could be defined were related to the overall speed, thereby providing an ability to assess stair running techniques.

## 2. Materials and Methods 

We tested 11 healthy volunteer subjects (three female, eight male; age: 25.6 ± 3.7 years; mean ± SD). The University of Michigan IRB approved the study and all subjects provided informed consent. Subjects were instructed to run up a long staircase at maximum speed, without skipping treads. After pausing for approximately ten seconds, the subjects ran down the same flight of stairs at maximum speed returning to the starting position, again without skipping treads. The staircase provided 16 strides total during the steady state (eight left and eight right). Subjects were not instructed which foot to begin stepping with for the task. The staircase rise height was 18 cm and the depth was 30 cm.

**Figure 1:**
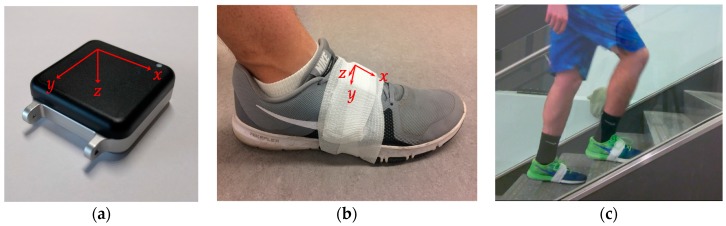
IMU data logger setup: (**a**) APDM IMU device showing the IMU sensor axes; (**b**) IMU attached to the shoe showing the IMU frame axes convention used in this paper; and (**c**) video stills showing the IMUs mounted on a subject's shoe climbing stairs ascent.

The subjects wore two IMU data loggers (Opals, APDM, Portland OR, USA; 128 Hz sampling, ±6 *g* acceleration, ±2000 deg/s angular rate), one mounted on each shoe affixed using athletic tape to the top of the laces (see Figure 1). The IMUs measure and store three components of linear acceleration $\left(a_{f}=[a_{x},\;a_{y},\;a_{z}]\right)$ from the on-board accelerometer and three components of angular velocity $\left(\omega_{f}=\left[\omega_{x},\;\omega_{y},\;\omega_{z}\right]\right)$ from the on-board angular rate gyro, both relative to the sensor-fixed axes (x,y,z). These sensor axes define the IMU frame of reference. We also define a navigation frame that overlaps with the IMU frame during initialization. The navigation frame remains affixed to the world during the experiment, while the IMU frame moves with the subject’s foot. Since the IMU sensor measurements are relative, there is no need to follow a strict anatomical calibration. However, since the IMU reference frame determines the navigation frame during initialization, it is advisable to approximately align the IMU axes to the desired navigation frame (see Figure 1). In-depth explanations of how (strap-down) IMUs are used, particularly for navigation applications, are provided in [24,25]. Major results from this field that we employ are summarized below.

### 2.1. Orientation Estimation

Estimating the foot trajectory from IMU data begins with first estimating the orientation of the foot-mounted IMU. For this purpose, we choose a quaternion (q) representation of the IMU orientation. Unlike the more common Euler angle representation that suffers from gimbal-lock, the quaternion representation readily describes any arbitrary sequence of rotations [26]. Quaternions represent an orientation as a rotation angle about a rotation axis. Thus, quaternions are defined using four parameters, one defining the angle of rotation and three defining the axis of rotation (e.g., three direction cosines). The four quaternion parameters satisfy the differential equation:

(1) $$\dot{q}=\frac{q\circ \Omega }{2}$$

(2) $$\Omega =\left[0,\;\omega_{x},\;\omega_{y},\;\omega_{z}\right]$$

in which the operator ∘ denotes quaternion multiplication [25,27] and Ω is a four-element vector containing the aforementioned measured angular velocity components $\left(\omega_{f}\right)$. Thus, the solution of (1) using the measured Ω yields the gyro-estimated orientation of the IMU as a function of time.

The gyro-estimated orientation will inevitably drift due to sensor errors, including bias drift, scale factor errors, and acceleration sensitivity. Our algorithm fuses the gyro-estimated orientation with accelerometer-estimated tilt angles from vertical (roll and pitch). This is achieved using a Kalman filter [28,29]. When the IMUs are mounted on the feet, the foot and the attached IMU are essentially stationary during specific time periods $\left(t_{s}\right)$ for the stance phase of each stride. The stationary periods are detected by observing the gyroscope and accelerometer measurements (see [14] Section 2.1 for more information about how $t_{s}$ is determined). During stationary periods, the accelerometer measures the components of gravity (G) along each sense axis. These measures are used to form accelerometer-estimated roll and pitch angles $\left(z=\left[\phi_{a},\theta_{a}\right]\right)$ per:

(3) $$\phi_{a}=\sin^{-1}\left(\frac{a_{x}}{G}\right)$$

(4) $$\theta_{a}=-\sin^{-1}\left(\frac{a_{y}G}{\cos\phi_{a}}\right)$$

**Figure 2:**
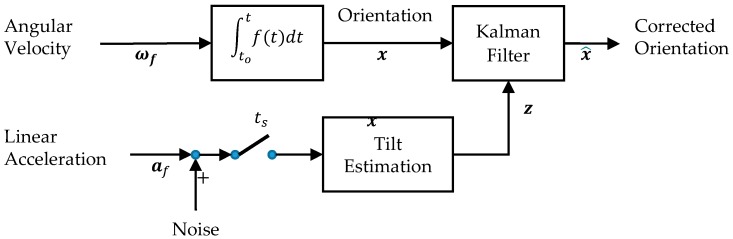
Angular velocity components measured by the gyroscope are integrated once to obtain orientation estimates x. The accelerometer components are used to estimate tilt (roll and pitch) during stationary periods $t_{s}$. The Kalman filter bounds the tilt errors by fusing the gyro-based orientation and accelerometer-based tilt to establish the “corrected orientation” $\hat{x}$.

Next, we use the gyroscope-estimated quaternion (q) value to calculate the equivalent Euler angles $\left(x=\left[\phi_{g},\theta_{g},\psi_{g}\right]\right)$, which also includes the estimated yaw angle $\left(\psi_{g}\right)$ (that is temporarily ignored as it cannot be detected from the accelerometers). The Kalman filter states $\left(\hat{x}=\left[\hat{\phi },\hat{\theta }\right]\right)$ are estimated as a combination of the gyroscope-based and accelerometer-based tilt estimates. We assume that all gyroscope error contributions and accelerometer-based tilt errors can be modeled as zero mean Gaussian noise. Since the process and measurement covariance errors are sensor-dependent only, once the Kalman filter is tuned the parameters are valid for all participants. The updated state is then converted back to its corresponding quaternion value. Figure 2 illustrates a block diagram of this orientation estimation algorithm.

### 2.2. Foot Trajectory Estimation

The resulting orientation estimates are used to resolve the foot IMU frame-acceleration components $\left(a_{f}\right)$ into the navigation frame acceleration components $\left(a_{n}\right)$. The z-axis component of the resultant world-referenced acceleration $a_{n}$ will be affected by gravity G:

(5) $$a_{w}=r_{f}^{n}a_{f}+G$$

in which $r_{f}^{n}$ is the rotation matrix from the foot IMU frame to the navigation frame as computed from the quaternion q [24,25]. Next, integrating $a_{n}$ once and then twice yields the foot IMU velocity (v) and position (p):

(6) $$v=v_{o}+\int_{t_{o}}^{t}a_{n}dt$$

(7) $$p=p_{o}+\int_{t_{o}}^{t}vdt$$

Since the experiment starts with a stationary phase, the initial velocity $\left(v_{o}\right)$ is zero and at a position $\left(p_{o}\right)$ also designated as zero. However, this can be generalized to a non-zero initial velocity or position for applications that require such. Examples of software implementations of (1)–(7) are found in [30,31].

**Figure 3:**
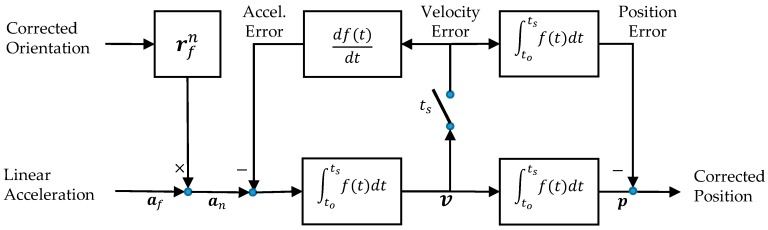
The accelerometer measurements are resolved in the world coordinate frame using the corrected orientation. The resultant accelerations are integrated twice to determine velocity and position. During stationary periods $t_{s}$, any remaining velocity is considered an error and its value is used to reset the position and acceleration errors.

The velocity estimated from (6) is often polluted by residual drift error (deriving from both the gyro and the accelerometer) which leads to (often slowly varying) velocity errors. The velocity drift error can be estimated and (approximately) eliminated using the following procedure. During the stationary times $\left(t_{s}\right)$ any remaining estimated velocity during these times can be assumed to be caused by drift error. These velocity errors are used to correct both the velocity (6) and position (7) estimates using an algorithm known as the Zero velocity UPdaTe (ZUPT). A block diagram for the ZUPT algorithm is illustrated in Figure 3 and further details of its implementation can be found in [14,22].

### 2.3. Elevation Correction

**Figure 4:**
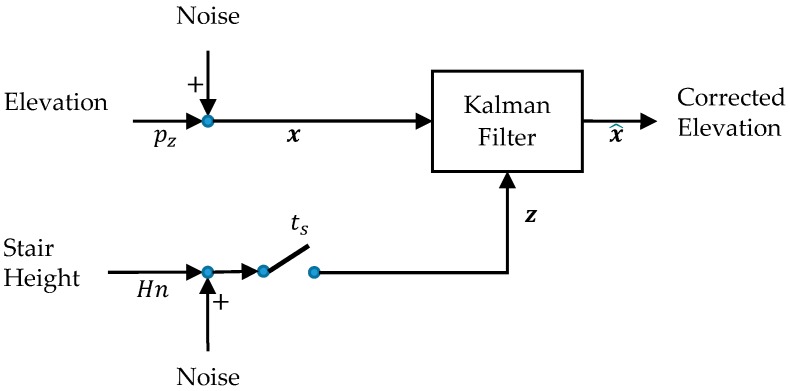
A Kalman filter makes foot elevation corrections using the known step height (riser), during each stationary time $t_{s}$.

Since the riser (step height) and tread (step depth) dimensions of the stairs are known, we add an additional correction to the position estimate. In particular, we designed a single-state Kalman filter that makes corrections to the IMU-derived vertical foot position $\left(x=\left[p_{z}\right]\right)$ knowing the riser height (H) and the number of steps (n) to yield an elevation observation per footfall (z=[Hn]). The filter makes corrections to its state $\left(\hat{x}=\left[\hat{p_{z}}\right]\right)$ whenever the foot reaches a new tread during the stationary time $\left(t_{s}\right)$. The filter assumes that the state and observation are both affected by uncorrelated white noise. A block diagram showing this filter is illustrated in Figure 4. Finally, we apply a linear interpolation in order to provide backward corrections to obtain the complete foot trajectory for each stride.

### 2.4. Gait Timing Variables

We used a wavelet analysis to establish the beginning (foot-strike) and end (toe-off) of each foot/ground contact period [32]. This approach is effective at identifying gait events because when the foot strikes or leaves the ground, the acceleration and angular velocity signals contain significantly more high-frequency content than at other times of the gait cycle. The wavelet analysis is used to identify time points when the measured signals contain significant content above 20 Hz, corresponding to either foot-strikes or toe-offs. Foot-strike time $\left(t_{strike}\right)$ was defined as the time when the foot first contacts a tread. For running on stairs, the toe is more likely to contact the tread first (whereas, during flat-surface walking the heel contacts the ground first). The initial contact $t_{strike}$ estimation does not require it to be a heel or toe specifically. Toe-off time $\left(t_{off}\right)$ is defined as the time when the foot first loses contact with the tread. The durations of the major phases of the gait cycle are important indicators of stair-climbing performance. In particular, we consider the durations of: (1) the entire stride; (2) the stance phase; and (3) the swing phase. The stride time $t_{stride}$ is measured as the time it takes from one foot-strike to the next foot-strike of the same foot during steady state. The stance time $t_{stance}$ is the time difference between two consecutive foot-strike and toe-off events. The swing time $t_{swing}$ is the time difference between two consecutive toe-off and foot-strike events:

(8) $$t_{stride}=\Delta t_{strike}$$

(9) $$t_{stance}=t_{off}-t_{strike}$$

(10) $$t_{swing}=t_{stride}-t_{stance}$$

We calculate the percentage of time that the subjects remain in the stance phase:

(11) $$t_{ps}=100\times \frac{t_{stance}}{t_{stride}}$$

Assuming left-right gait symmetry [33], a $t_{ps}$ value larger than 50% indicates the existence of a double support phase (when both feet are in contact with the ground simultaneously).

### 2.5. Gait Kinematic and Kinetic Variables

Beyond the timing of gait events, our approach provides the full trajectory and orientation of the feet, which are useful for understanding stair running performance. Foot clearance (c) is defined as the foot height $\left(p_{z}\right)$ difference between the times of the local maximum $\left(t_{max}\right)$ and minimum $\left(t_{min}\right)$ around foot-strike:

(12) $$c=p_{z}\left(t_{max}\right)-p_{z}\left(t_{min}\right)$$

**Figure 5:**
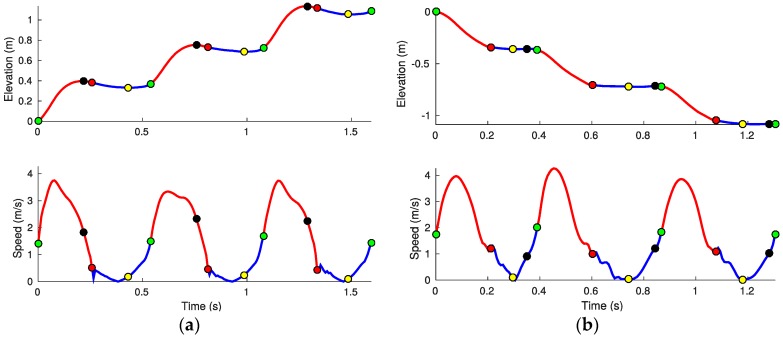
Estimated foot trajectory and speed for running over three treads during ascending (**a**) and descending (**b**). Close up of a steady state running gait showing the major stride events times: toe-off $t_{off}$ (green dots), foot strike $t_{strike}$ (red dots), maximum elevation $t_{max}$ (black dots), minimum elevation $t_{min}$ (yellow dots); and gait phases: stance phase $t_{stance}$ (blue curves) and swing phase $t_{swing}$ (red curves).

In particular, for every stride we identify the local minimum foot height $\left(t_{min}\right)$ after the $t_{strike}$ and before $t_{off}$. For stair ascending, $t_{max}$ is defined as the time when the local maximum foot height occurs just prior to foot-strike (swing phase) while, for stair descending, it is identified after the foot strike and, in most cases, before toe-off (stance phase). Examples showing the typical distribution of local minimum and maximum times in the different gait cycles for stair running (both ascending and descending) are shown in Figure 5. One interpretation of the clearance, c is that it indicates how subjects minimize tripping risk as they plan for advancing to the next step (i.e., larger value of c could imply a more careful foot trajectory planning that provides a safer margin to clear the steps).

The estimated foot IMU velocity (6) is used to compute a proxy for the foot kinetic energy per unit of mass kem per using the following formulation:

(13) $$kem=\frac{k}{m}=\;\frac{{\bar{\left|v\right|}}^{2}}{2}$$

where $\bar{\left|v\right|}$ denotes the average magnitude of the foot speed calculated over the duration of every stride $t_{stride}$ (8).

During stair running, the foot rotates with the majority of rotation manifesting in changes in pitch θ. We estimate the “bounce angle” $\theta_{bounce}$ as the angular displacement in pitch from foot-strike to toe-off as follows:

(14) $$\theta_{break}=\left|\theta \left(t_{strike}\right)-\;\theta \left(t_{min}\right)\right|$$

(15) $$\theta_{prop}=\left|\theta \left(t_{off}\right)-\;\theta \left(t_{min}\right)\right|$$

(16) $$\theta_{bounce}=\theta_{break}+\theta_{prop}$$

Here, the “braking angle” $\theta_{break}$ is computed as the change in foot pitch from the contact time $t_{strike}$ until the foot reaches its minimum elevation during the stance phase. The “propulsion angle” $\theta_{prop}$ is computed as the change in foot pitch from the time of minimum elevation until toe-off $t_{off}$. The resulting bounce angle could be related to ankle stiffness used during propulsion [34], which implicates performance outcomes [35] (i.e., stiffer ankles limit the time delay, or, “give” in the transmission of forces up the kinetic chain) or risk for injury [36].

By estimating the impulse between foot-strike and toe-off events, we also estimate the foot vertical ground reaction force per unit of mass gfm per:

(17) $$gfm=\frac{f_{z}}{m}=\;\frac{\Delta v_{z}}{\Delta t}$$

(18) $$\Delta v_{z}=v_{z}\left(t_{off}\right)-v_{z}\left(t_{strike}\right)$$

where the time increment Δt equals the $t_{stance}$ (9).

### 2.6. Statistical Analysis

In our analysis, we eliminated the first and the last step from each stair run, as we considered them to be transition steps that differ from the approximately steady state stepping that is the focus of our study. We also assumed left-right foot symmetry and pooled these data within the statistical analysis. This study does not consider or use the anthropometric characteristics of the participants.

To evaluate how the gait timing, kinematic, and kinetic parameters were related to the stride times (speed), we performed a simple linear regression for each relationship to determine: the R-squared value (*R*^2^) to quantify the variation explained by the relation; the slope of the relation (b) between the metric of interest and the stride time; and the statistical significance of the slope $\left(p_{b}\right)$. The simple linear regression assumptions of normality and constant variance of the residual were assessed using the Lilliefors test and Engle’s Auto Regressive Conditional Heteroskedasticity (ARCH) test, respectively. When these conditions were not met, a transformation of the variables was performed and the simple linear regression was fit to the transformed variables to assess if the relationship trends were consistent. Comparison of the variation between $t_{swing}$ and $t_{stance}$ was assessed using an F-test. We use a two-sample *t*-test to compare the ascending and descending conditions for the $t_{ps}$, c, and $\theta_{bounce}$ variables. Finally, we use a one-sample t-test to determine if gfm was different than zero.

## 3. Results and Discussion

Figure 5 shows an example of the estimated foot elevations and velocity magnitudes against time for a subject running while ascending (Figure 5a) and descending (Figure 5b) the stairs. The trajectories illustrate several steady state strides with labelled times for foot-strike, toe-off, maximum elevation, minimum elevation, and gait phases.

**Figure 6:**
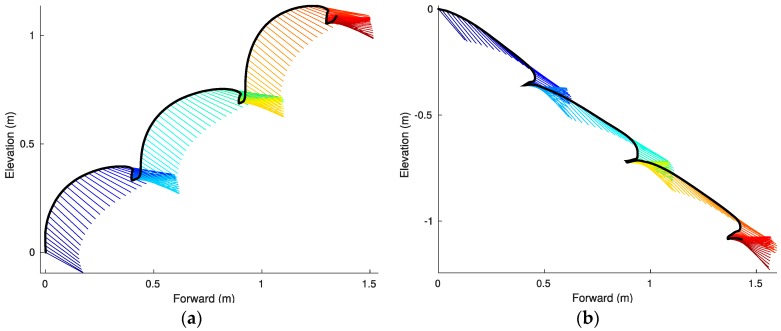
Foot trajectory (black curve) and pitch angle θ (colored lines) for ascending (**a**) and descending (**b**) stairs. The colors distinguish the distinct gait cycles across successive treads.

The above algorithm yields estimates of the full (three-dimensional) trajectories, as well as (three-dimensional) foot orientation angles. Figure 6 presents a foot trajectory in space (elevation plotted versus forward position) as well as the foot pitch angle and for the same sample steps considered in Figure 5.

**Figure 7:**
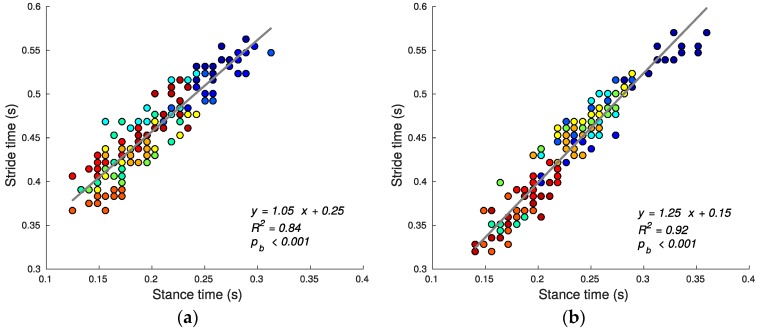
Stance $t_{stance}$ and stride time $t_{stride}$ relationship for ascending (**a**) and descending (**b**) stairs. Each dot represents one stride, and each color represents one subject. Overall speed is largely determined by the stance phase.

**Figure 8:**
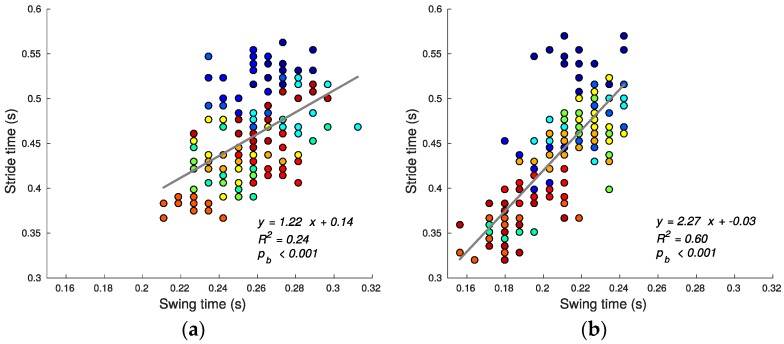
Swing $t_{swing}$ and stride time $t_{stride}$ relationship for ascending (**a**) and descending (**b**) stairs. The greater correlation during stair descent indicates that subjects likely generate speed gains during the swing phase.

**Figure 9:**
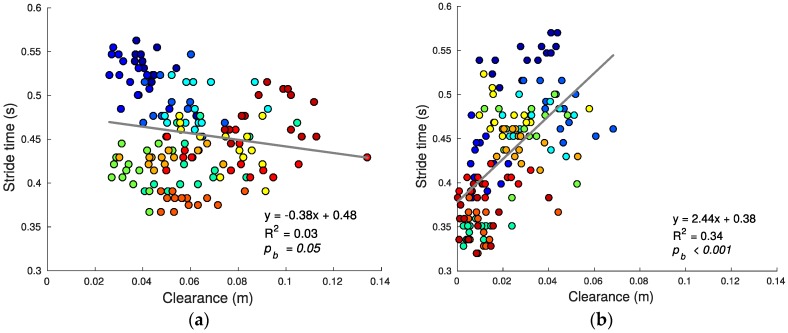
Foot clearance c and stride time $t_{stride}$ relationship for ascending (**a**) and descending (**b**) stairs. Descent is accomplished with an overall smaller clearance relative to ascent.

**Figure 10:**
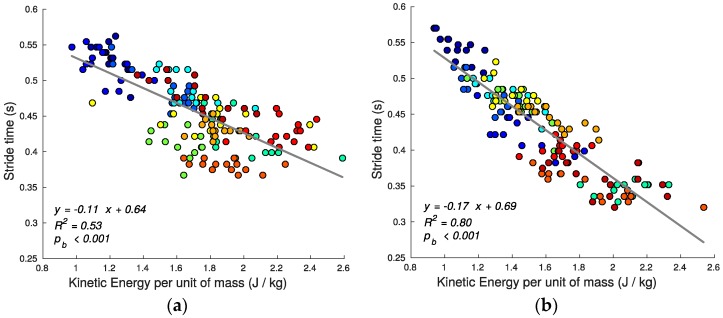
Kinetic energy per unit of mass kem and stride time $t_{stride}$ relationship for ascending (**a**) and descending (**b**) stairs. In stair ascent, a fraction of the kinetic energy is consumed in order to safely clear the nose of the treads.

**Figure 11:**
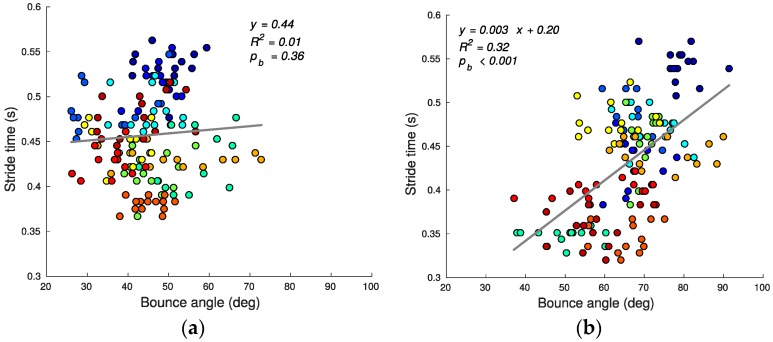
Bounce angle $\theta_{bounce}$ and stride time $t_{stride}$ correlation for ascending (**a**) and descending (**b**) stairs. Lower bounce angle during stair ascent is related to impulsive motion.

**Figure 12:**
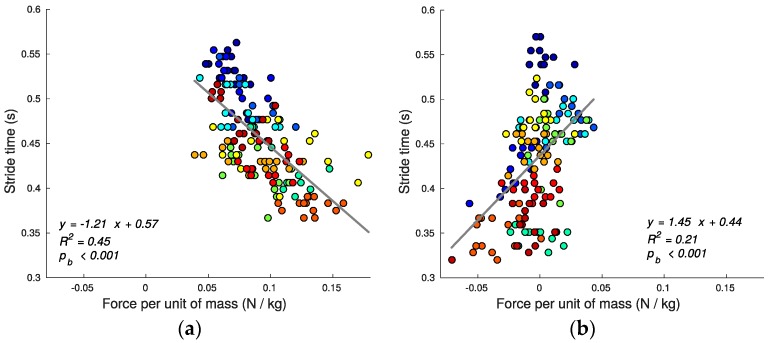
Vertical ground reaction force per unit of mass gfm and stride time $t_{stride}$ relationship for ascending (**a**) and descending (**b**) stairs. Stair ascent employs significantly larger impulses relative to descent.

Using speed alone as the criterion, stair running performance can then be quantified by the stride time (shorter average stride time predicting greater average speed since step lengths are defined/constrained by the stairs geometry). Figure 7, Figure 8, Figure 9, Figure 10, Figure 11 and Figure 12 compare the individual stride times (vertical axis) against all other metrics, including the additional gait timing, kinematic, and kinetic variables defined above (horizontal axes). In these figures, each dot represents one stride during steady state, and each color represents one subject. We also provide the equation of the linear fit, *R*^2^, and $p_{\beta }$ for each relation.

### 3.1. Gait Timing Variables

Our data analysis shows that in either direction (stair ascent or decent), the stride time $t_{stride}$ was mainly predicted by the stance time $t_{stance}$ as measured by high correlation (*R*^2^ value for ascent 0.84, $p_{b}$ < 0.001; *R*^2^ for descent 0.92, $p_{b}$ < 0.001); refer to Figure 7. Thus, shorter $t_{stance}$ values are strong predictors of overall speed (shorter stride times) during both stair ascent and decent.

Due to the restrictions imposed by the stair design, subjects are relatively constrained during the swing phase. Regardless of speed, the feet must travel approximately the same distance. Thus, one expects less variation in $t_{swing}$ than in $t_{stance}$. This expectation is supported by the smaller standard deviation of $t_{swing}$ (SD for ascent 0.020 s, for descent 0.022 s) compared to that for the $t_{stance}$ (SD for ascent 0.044 s, for descent 0.049 s) across all subjects (F(153, 153) = 4.67, *p* < 0.001 for ascent; F(153, 153) = 5.06, *p* < 0.001 for descent). During stair ascent, subjects provide just enough speed to reach the next tread, since otherwise they risk missing, tripping, or overshooting, making the task either dangerous or inefficient. As a result, there is a lower correlation between $t_{stride}$ and $t_{swing}$ during ascent (*R*^2^ value 0.24) (see Figure 8a). During stair descent, however, subjects have more freedom to choose higher speeds during the swing phase by using their muscles to break less, as shown by the higher correlation between $t_{swing}$ and $t_{stride}$ for stair descent (*R*^2^ value 0.60) (see Figure 8b). This gain in speed comes at the expense of having to accommodate for higher foot-strike impacts and increasing fall risk.

Finally, we observe that when running downstairs, subjects do so more carefully, as manifested in a greater (t(306) = −15.65, p < 0.001) percentage of time $t_{ps}$ (11) that the subjects remain in the stance phase while descending (ascending: 44.3 ± 10.8%, descending: 53.4 ± 13.5%; mean/SD). We conclude that $t_{stride}$ is highly correlated with $t_{stance}$ and therefore speed is determined largely by the ability of the subjects to generate enough impulse to reach the next step in the shortest period of time.

Table 1 presents a summary of the gait timing variables. To summarize, both $t_{stance}$ and $t_{swing}$ have significant relationships to speed. However, $t_{stance}$ shows the highest correlation, indicating the potential to be a better predictor.

### 3.2. Gait Kinematic and Kinetic Variables

While the estimated slope between $t_{stride}$ (speed) and foot clearance c for ascent was significant, there is a negligible relationship between these variables as seen by the low *R*^2^ value (*R*^2^ for ascent 0.03, $p_{b}$ = 0.05). There was a significant linear relation for descent (*R*^2^ = 0.34, $p_{b}$ < 0.001) (see Figure 9). During descent, subjects clear the steps with a smaller average clearance relative to ascent (ascending: 0.06 ± 0.02 m, descending: 0.02 ± 0.02 m; mean ± SD; t(306) = 17.49; p < 0.001), in some cases by rolling the foot on the nose of the tread as they transition to the next tread. Smaller average clearance enables the foot to follow a more linear trajectory (see Figure 6), which can be more energy efficient as explained in the next section.

A significant linear relationship between the foot kinetic energy kem (13) and $t_{stride}$ exists (*R*^2^ for ascent: 0.53, $p_{b}$ < 0.001; *R*^2^ for descent: 0.8, $p_{b}$ < 0.001) (see Figure 10), with faster subjects exhibiting higher kinetic energy. During stair ascent, a fraction of the kinetic energy is consumed just to clear the nose of the steps safely and, as a result, the foot describes a parabolic trajectory (see Figure 6a) in strong contrast with the linear trajectory exhibited during descent (see Figure 6b).

Example variations in the pitch angle during stair running are illustrated in Figure 6. The pitch variations $\theta_{bounce}$ (14)–(16) do not have a linear relationship with $t_{stride}$ (Figure 11) during ascending (*R*^2^ for ascent: 0.01, b = 0, $p_{b}$ = 0.36) and have a moderate relationship during descending (*R*^2^ for descent: 0.32, b = 3 × 10^−3^, $p_{b}$ < 0.001). The average bounce angle during ascent is smaller than the average bounce angles during descent (ascending: 45.0 ± 9.2 deg, descending: 66.2 ± 10.4 deg; mean ± SD; t(306) = −19.029; p < 0.001). It is noteworthy that during the stair ascent smaller bounce angles are indicative of an increase in ankle stiffness which, in turn, increases vertical velocity [35].

We calculated foot vertical ground force gfm using (17), and determined that there was moderate correlation between $t_{stride}$ and gfm for both ascent and descent (*R*^2^ for ascent: 0.45, $p_{b}$ < 0.001; *R*^2^ for descent: 0.21, $p_{b}$ < 0.001) (see Figure 12). The gfm mean value shows that ascending stairs requires generating a non-zero reaction force (0.09 ± 0.03 N/kg; mean ± SD; t(153) = 40.97, *p* < 0.001), whereas the descending force was not statistically different from zero (0.0 ± 0.02 N/kg, mean ± SD; t(153) = −1.96, *p* = 0.052). This suggests distinct mechanisms for running on stairs with ascending requiring changes in momentum (impulses), while descending requires maintaining momentum. Ascending stairs requires generating the necessary force needed to propel the body upwards and forwards; conversely, during descending the muscles have less resistance (as supported by the increase in bounce angle) allowing gravity to do the work.

The kinematic and kinetic variables are summarized in Table 2. In summary, we determine that clearance, c, is only correlated to speed during stair descent. We found that some kem is lost during stair ascent because of the foot parabolic trajectory required to clear safely the steps. The foot angle $\theta_{bounce}$ shows ankle stiffness during stair ascent versus compliance during stair descent. The effect of $\theta_{bounce}$ is also evident in ground forces gfm being large for stair ascent and negligible for stair descent.

For every simple linear regression relation, the assumptions of normality and constant variance of the residuals were tested (see Table 1 and Table 2). For the cases that did not meet the assumptions, we used data transformation algorithms to correct for distribution skewness as described in [37,38] and verified the significance of the relationships when assumptions were met. To facilitate the interpretation of the measures, we presented the relationships for the variables prior to transformation. It is important to note that while the slopes may differ with the transformed variables, the direction and significance of the relationship would not be expected change the results presented.

Finally, it is important to note that the sensors that we use have limited operational range that may influence some of the outcomes, in particular the vertical acceleration during the foot-strike could be underestimated. We believe that the final effect of this limitation in our calculations is small due to the short duration of this event, the elevation correction that we perform, and our stride-by-stride basis analysis instead of the whole trajectory.

## 4. Conclusions

This paper presents a method for understanding the task of running on stairs (both ascending and descending) from data harvested from foot-mounted IMUs. This understanding derives from an algorithm that estimates the foot velocity and trajectory while correcting for sensor drift errors using the ZUPT technique together with a known stair riser height. In studies of human mobility outside of a controlled experimental setup, during which stair height may not be known to the researchers, implementing a “standard” step height correction may still assist in calculating stride metrics. Timing, kinematic, and kinetic variables are proposed as metrics of stair running performance. Results on human subjects reveal that stair running speed is largely controlled by the stance phase, as opposed to the swing phase. An approximate measure of foot kinetic energy illustrates greater foot energy economy during descent versus ascent, which also follows from the near-linear foot trajectory during descent versus the parabolic path during ascent. The IMU-derived estimates for foot clearance may have future use in assessing trip/fall risks while the IMU-derived estimates of ground reaction and bounce angle may have future use in assessing injury potential.

## Figures and Tables

**Table 1:** Gait cycle timing variables for running while ascending and descending stairs.

Direction	$t_{stride}$ vs. $t_{stance}$ *R*^2^/b	$t_{stride}$ vs. $t_{swing}$ *R*^2^/b	$t_{stance}$ SD (s)	$t_{swing}$ SD (s)	$t_{ps}$ Mean ± SD (%)
Ascent	0.84/1.05	0.24/1.22 ^†^	0.044	0.020	44.3 ± 10.8
Descent	0.92/1.25 ^†^	0.60/2.27 ^†^	0.049	0.022	53.4 ± 13.5

^†^ Does not meet constant variance assumption.

**Table 2:** Kinematic and kinetic variables for running while ascending and descending stairs.

Direction	$t_{stride}$ vs. c *R*^2^/b	$t_{stride}$ vs. kem *R*^2^/b	$t_{stride}$ vs. $\theta_{bounce}$ *R*^2^/b	$t_{stride}$ vs. gfm *R*^2^/b	c Mean ± SD (m)	$\theta_{bounce}$ Mean ± SD (deg)	gfm Mean ± SD (N/Kg)
Ascent	0.03/−0.38 ^‡,^*	0.53/−0.11 ^‡^	0.01/0.0 ^†,^*	0.45/−1.21	0.06 ± 0.02	45.0 ± 9.2	0.09 ± 0.03
Descent	0.34/2.44 ^†^	0.80/−0.17 ^‡^	0.32/3 × 10^−3^	0.21/1.45	0.02 ± 0.02	66.2 ± 10.4	0.0 ± 0.02

* b Not statistically significant. ^†^ Constant variance assumption not met. ^‡^ Normality assumption not met.
